# Excellent Light Confinement of Hemiellipsoid- and Inverted Hemiellipsoid-Modified Semiconductor Nanowire Arrays

**DOI:** 10.1186/s11671-018-2659-2

**Published:** 2018-08-15

**Authors:** Xinyu Chen, Jiang Wang, Pengfei Shao, Qiming Liu, Dequan Liu, Qiang Chen, Yali Li, Junshuai Li, Deyan He

**Affiliations:** 1 National and Local Joint Engineering Laboratory for Optical Conversion Materials and Technology, Key Laboratory of Special Function Materials and Structure Design of the Ministry of Education, and School of Physical Science and Technology, Lanzhou University, 222 South Tianshui Road, Lanzhou, 730000 China; 2 Institute of Electromagnetics and Acoustics, Department of Electronic Science, and Fujian Provincial Key Laboratory of Plasma and Magnetic Resonance, Xiamen University, Xiamen, 361005 China

**Keywords:** Optical properties, Photovoltaic, GaAs, Solar cells, Physical optics

## Abstract

In this paper, we introduce hemiellipsoid- and inverted hemiellipsoid-modified semiconductor nanowire (NW) optical structures, and present a systematic investigation on light management of the corresponding arrays based on GaAs. It is found that the modification makes well utilization of light scattering and antireflection, thus leading to excellent light confinement with limited effective thickness. For example, 90% and 95% of the incident photons with the energy larger than the bandgap energy can be trapped by the inverted hemiellipsoid-modified NW arrays with the effective thicknesses of only ~ 180 and 270 nm, respectively. Moreover, excellent light confinement can be achieved in a broad range of the modification height. Compared to the corresponding array without top modification, spatial distribution of the photo-generated carriers is expanded, facilitating carrier collection especially for the planar *pn* junction configuration. Further investigation indicates that these composite nanostructures possess excellent omnidirectional light confinement, which is expected for advanced solar absorbers.

## Background

Solar electricity based on the photovoltaic (PV) effect has made a remarkable progress in the past decades,and is gradually changing the global energy structure [1–10]. To meet the continuously increasing demand of PV electricity, large-scale deployment of PV modules is urgent, and meanwhile restricted by the relatively high price, which is mainly related to high material costs of the market-dominated PV products based on crystalline silicon wafers [11–20]. Although thin film-based PV devices have the huge potential for material cost reduction, poor light absorption due to the limited optical thickness is a big concern and needs to be addressed by introducing light management structures, such as antireflection coatings and/or substrate texturing, which would result in the extra cost [21–27].

Different from the traditional planar structures, nanostructured semiconductor solar absorbers possess superior properties in light management and photo-generated carrier collection and thus exhibit huge potential in application of high performance-to-cost optoelectronic devices including solar cells and photodetectors [28–36]. Thanks to the extensive efforts dedicated by the related researchers, various semiconductor nanostructures such as nanowire (NW) [37–45], nanocone [46–50], nanopit [51–53], and nanohemisphere [54, 55] arrays have been introduced and investigated from both theoretical and experimental aspects. Effects of light management modes including modification of spatial refractive index for antireflection, leaky mode, guided longitudinal resonance, light scattering, and surface plasmon resonance on light trapping have been understood and emphasized with different weights for different nanostructures [56–61]. However, each individual light management mode cannot fulfill efficient light confinement in a broad spectral range, especially for solar cell applications. Accordingly, combination of different light management modes is necessary for full spectral absorption enhancement. Meanwhile, considering the concerns related to fabrication issues, e.g., high reproducibility at low cost, simple structure for light absorbers is required.

To realize more efficient light confinement with limited effective thickness for semiconductor NW arrays, top modification using hemiellipsoid and inverted hemiellipsoid structures is introduced and systematically investigated on the light management behaviors in this paper. Owing to the synergetic effect of effective antireflection and light scattering, light confinement is significantly boosted with reduced effective thickness as compared to the NW arrays without modification. For the case of GaAs NW arrays, 90% and 95% of the incident photons with the energy larger than the bandgap energy can be trapped by the inverted hemiellipsoid-modified NW arrays with the effective thickness of ~ 180 and 270 nm. Moreover, further study indicates that the related structures deliver excellent light confinement under oblique incidence.

## Methods

**Fig. 1:**
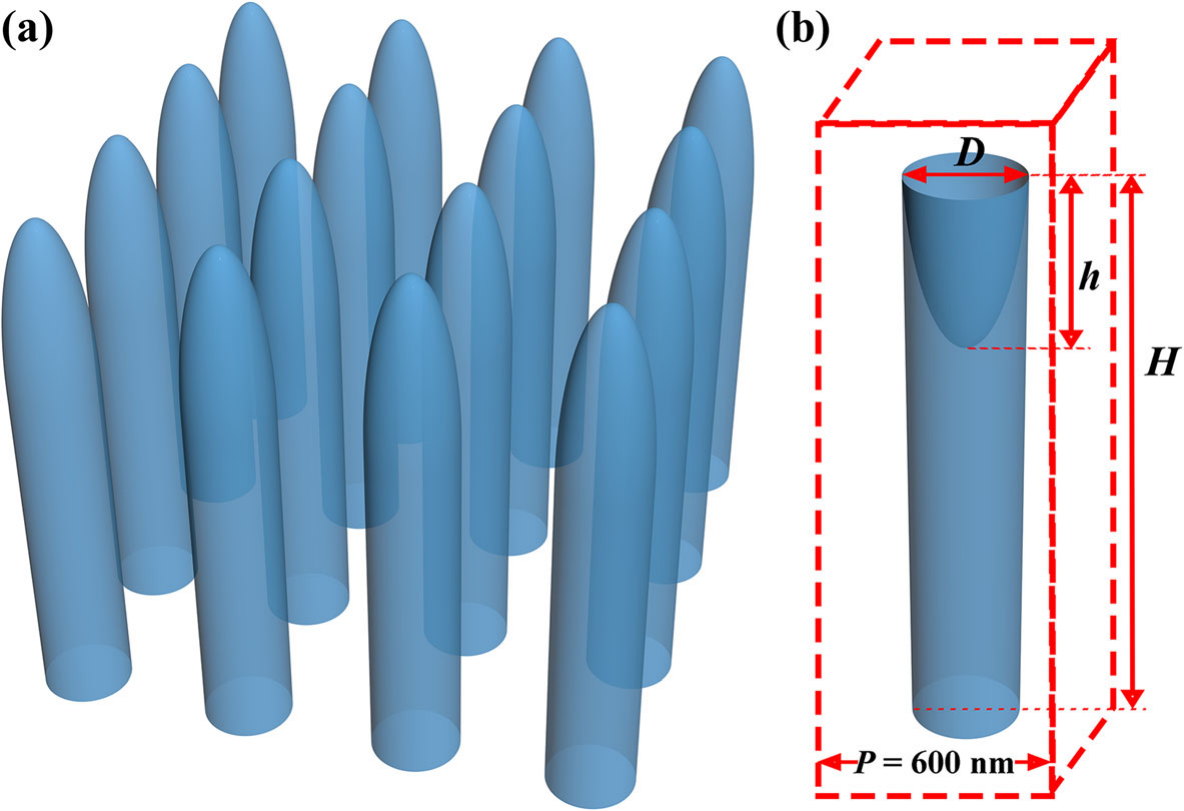
**a** Schematic of a hemiellipsoid-modified NW array, and **b** a unit of an inverted hemiellipsoid-modified NW array for optical simulations. The structural parameters investigated in this study are the nanowire diameter (*D*), total height (*H*), and modification height (*h*) as labeled

In this study, squarely arranged NW arrays (see Fig. 1a) with an optimized period of 600 nm [56, 62] are investigated under different structural parameters of the nanowire diameter (*D*), total height (*H*), and modification height (*h*), as labeled in Fig. 1b. To calculate the Maxwell’s equations and thus the energy flux distribution of the optical systems, a finite difference time domain method is employed. Periodic boundary conditions are applied onto the side walls of a unit to construct the related arrays, and meanwhile benefit saving of the calculation source and time. At the upper and bottom bounds of the unit, the perfect matching layer boundary is used to absorb all outgoing photons and thus to determine light reflection (*R*) and transmission (*T*). Then light absorption (*A*) is obtained following the relationship of *A* = 1–*R*–*T*.

In this paper, the representative semiconductor optoelectronic material, GaAs, is adopted for investigation. Considering the bandgap energy of 1.42 eV and the main energy region of the solar irradiation, optical behaviors in a spectral range of 300–1000 nm are investigated. To more quantitatively compare light trapping of the optical systems, normalized theoretical photocurrent density, ^N^*J*_ph_, is adopted [27, 63], which is defined as the ratio of the theoretical photocurrent density of the investigated structure to that (~ 32.0 mA/cm^2^ at AM 1.5G [64] illumination for GaAs) of an ideal absorber with the same bandgap energy both at an internal quantum efficiency of 100%.

## Results and Discussion

**Fig. 2:**
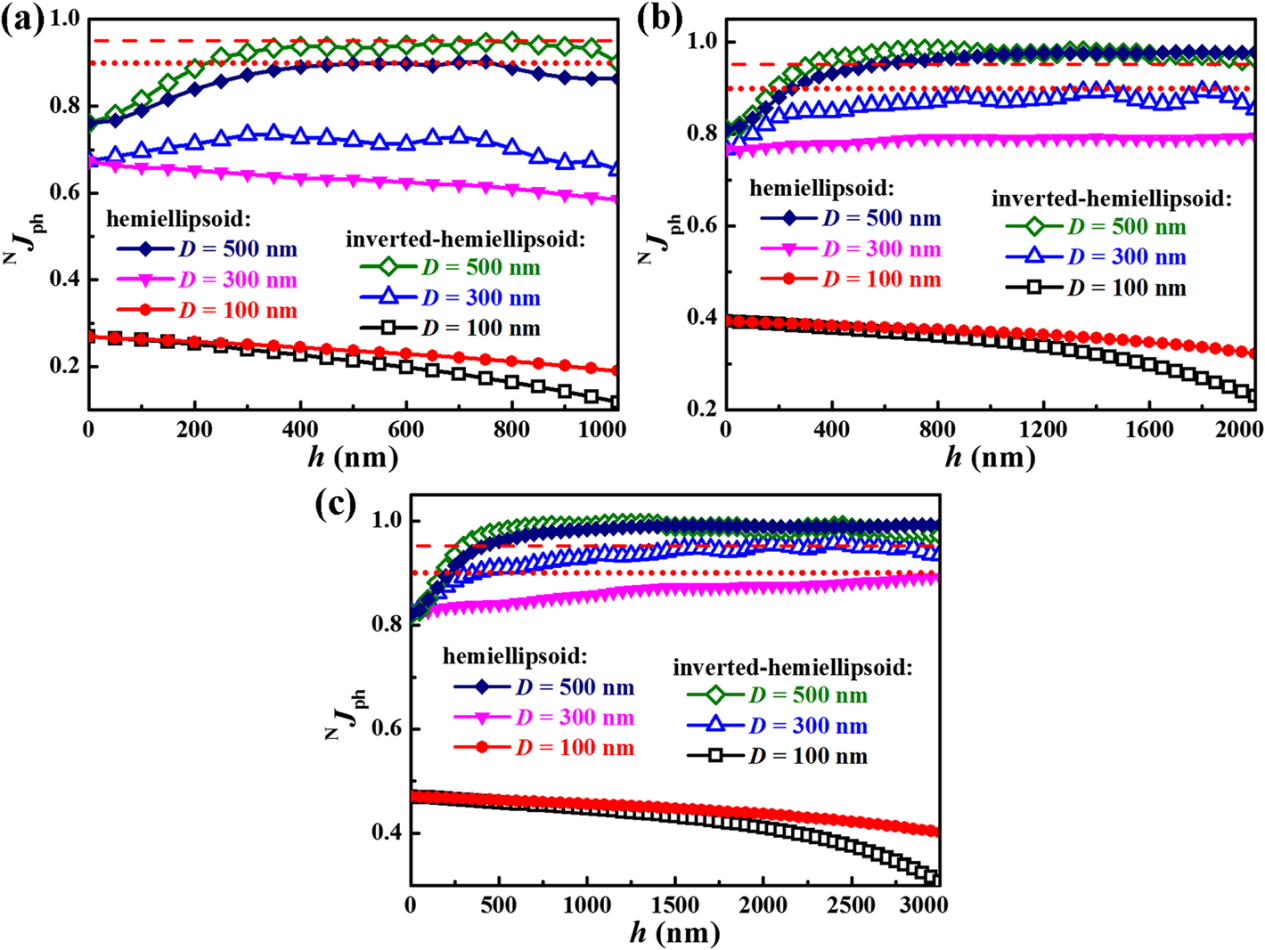
Normalized theoretical photocurrent density (^N^*J*_ph_) for the hemiellipsoid- and inverted hemiellipsoid-modified GaAs NW arrays as a function of the hemiellipsoid height (*h*) at different total heights of **a** 1000, **b** 2000, and **c** 3000 nm. The wire diameters (*D*) are 100, 300 and 500 nm. The red dot line and red dash line in each figure denote the values of ^N^*J*_ph_ of 0.90 and 0.95, respectively

Figure 2 summarizes ^N^*J*_ph_ as a function of *h* for the hemiellopsoid- and inverted hemiellipsoid-modified GaAs NW arrays with *H* of (a) 1000, (b) 2000, and (c) 3000 nm; and *D* of 100, 300 and 500 nm. One notes that ^N^*J*_ph_ for all arrays with *D* of 100 nm monotonously decreases with the increased *h*. However, for such arrays with larger *D* of 300 and 500 nm, enhanced light confinement can be generally observed after introducing top modification with appropriate sizes, except for the case of *D* = 300 nm and *H* = 1000 nm. Moreover, the thicker the NWs, the more remarkable enhancement of light confinement can be realized. It is notable that, as exhibited in Fig. 2a, ^N^*J*_ph_ of 0.90 and 0.95 can be achieved for the inverted hemiellipsoid modification with the effective thicknesses of only ~ 180 and 270 nm for the array with *D* = 500 nm, *H* = *h* = 1000 nm and the array with *D* = 500 nm, *H* = 1000 nm and *h* = 750 nm, respectively.

**Fig. 3:**
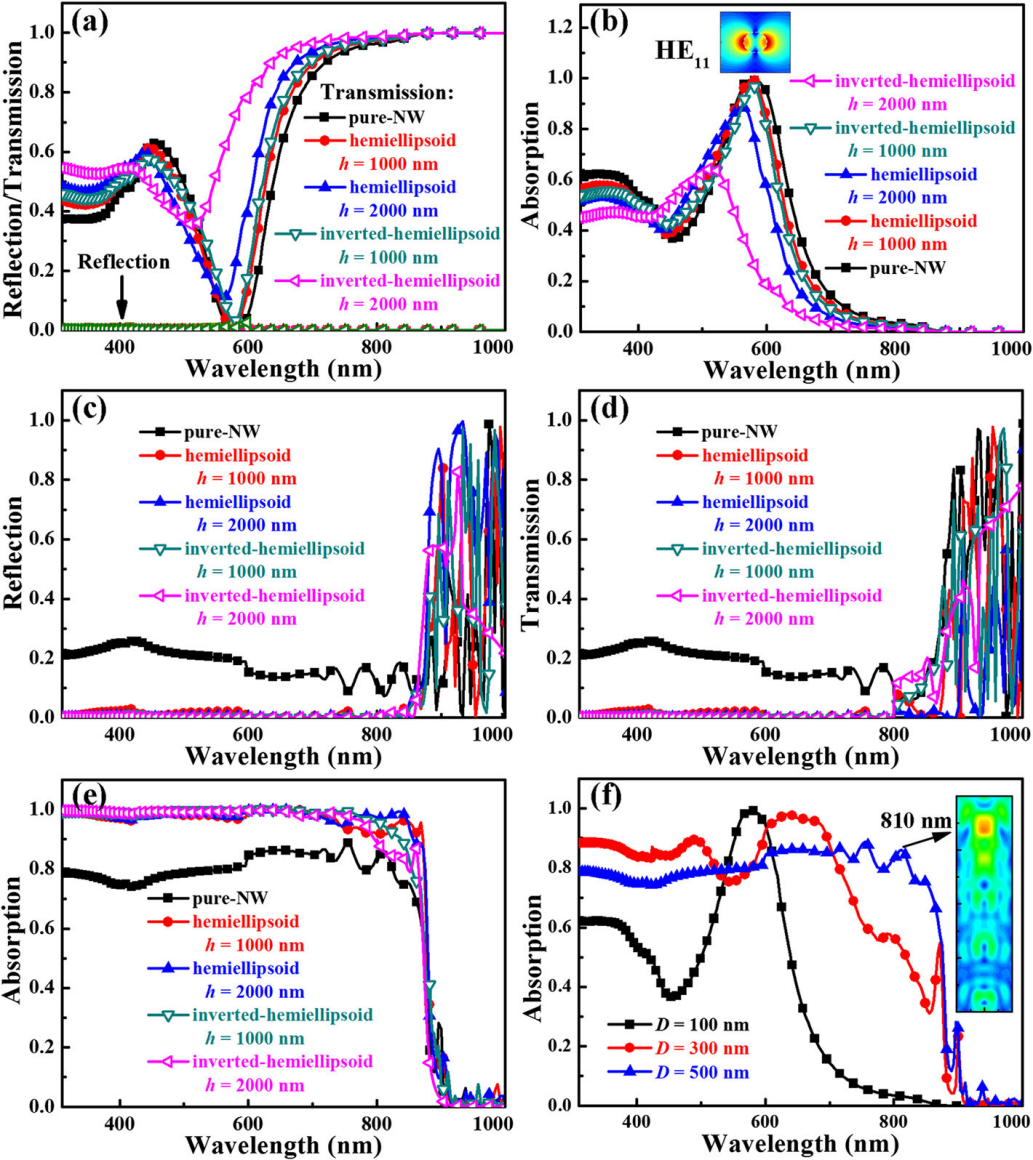
**a** Reflection/transmission and **b** absorption of the arrays of *H* = 2000 nm and *D* = 100 nm. **c** Reflection, **d** transmission, and **e** absorption of the arrays of *H* = 2000 nm and *D* = 500 nm. **f** Absorption of the pure NW arrays with *D* of 100, 300, and 500 nm and *H* = 2000 nm. The inset of **b** shows the electric field strength distribution of the HE_11_ mode, and the white dotted circle outlines the wire periphery. The inset of **f** exhibits the electric field strength distribution of the pure NW array with *H* = 2000 nm and *D* = 500 nm at the wavelength of 810 nm

It is well known that antireflection is an inherent function for NW arrays due to the reduced difference between refractive indices of the surrounding environment (normally air) and optical structure as compared to their flat wafer/film counterparts [27, 52]. However, antireflection does not consequently result in effective light absorption because of the possible enhancement of light transmission through the absorbers. In this study, the arrays with *D* of 100 nm possess the lowest filling ratio and thus the smallest effective refractive index. Although these arrays exhibit excellent antireflection, light transmission is significantly strong, especially in the long wavelength regime (see Fig. 3a), i.e., the high-density region of photons. Furthermore, as indicated in Fig. 3a, top modification has little contribution to antireflection, but leads to enhanced light transmission, thus making light absorption worse (see Fig. 3b), and resulting in the decrease of ^N^*J*_ph_ for the 100 nm NW diameter arrays. In addition, one notes that the main light confinement mechanism is the HE_11_ leaky mode (see the inset of Fig. 3b) for the NW arrays of *D* = 100 nm [65].

For the NW arrays with larger *D* of 300 and 500 nm, the filling ratio and thus the effective refractive index increase, and light reflection becomes evident, as shown in Fig. 3c. For these arrays, appropriate modification using both hemiellipsoid and inverted hemiellipsoid can remarkably reduce light reflection, thus enhances light absorption (see Fig. 3c and e). Moreover, it is evident that excellent light confinement can be achieved in a broad range of modification height, thus providing convenience for fabricating the related high-performance devices. For example, as exhibited in Fig. 2b, ^N^*J*_ph_ of 0.95 can be achieved for a 500 nm diameter NW array with inverted hemiellipsoid in range of 350–2000 nm or with hemiellipsoid in range of 600–2000 nm. However, excessive modification (i.e., *h* is too large) especially for the case using inverted hemiellipsoids would lead to significantly enhanced light transmission and reduced light absorption around the bandgap energy, as exhibited in Fig. 3d and e. Accordingly, the first increase and following decrease of ^N^*J*_ph_ is observed for the related NW arrays (see Fig. 2).

Figure 3f shows the absorption spectra of the pure NW arrays with *D* of 100, 300 and 500 nm, and *H* of 2000 nm. It is evident that light absorption edge shifts towards long wavelength, and meanwhile the main light management mechanism changes from leaky mode to light scattering as *D* increases. Moreover, for NWs with *D* of 500 nm, some absorption oscillations around 800 nm can be observed, which are attributed to the guided longitudinal resonances, as exhibited in the inset of Fig. 3f. It is known that as *D* increases, the threshold/longest wavelength that can form a guided longitudinal mode also increases [56, 57]. For long-wavelength light, the amplitude decay when propagating along the wire axis is relatively weaker than that of short-wavelength light because of the smaller absorption coefficient. If the wire length is not too long, the reflected wave from the NW bottom can interfere with the incoming wave to form the guided longitudinal resonances.

**Fig. 4:**
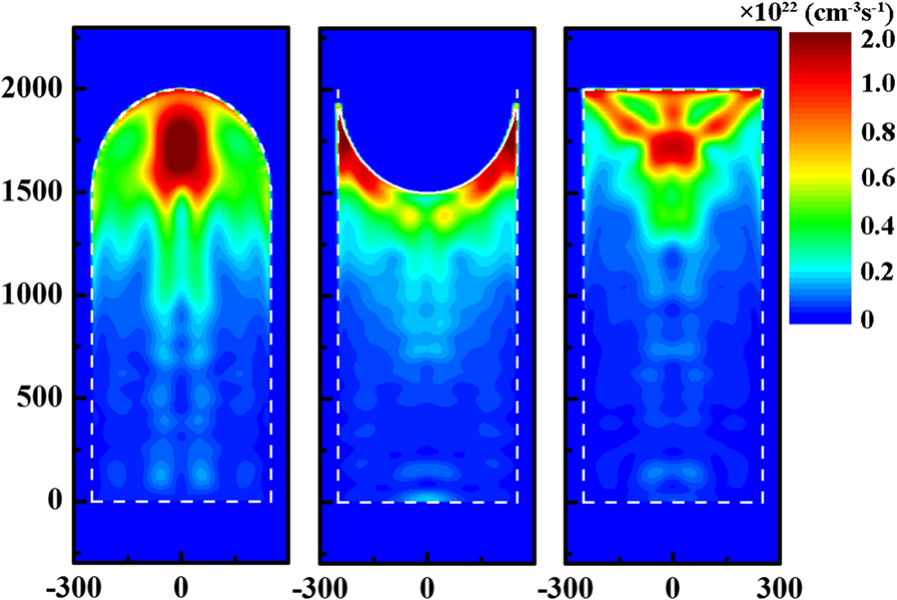
Spatial distribution of the photo-generated carrier generation rate at AM 1.5G illumination for the arrays (*H* = 2000 nm and *D* = 500 nm) top-modified by (left) hemiellipsoids (*h* = 500 nm) and (middle) inverted hemiellipsoids (*h* = 500 nm). The generation rate (right) in the pure NW array of *H* = 2000 nm and *D* = 500 nm is presented for comparison

To further understand influence of top modification on light management, spatial distribution of the carrier generation rate for the arrays (*H* = 2000 nm and *D* = 500 nm) modified by hemiellipsoids (*h* = 500 nm) and inverted hemielliopsoids (*h* = 500 nm) at AM 1.5G illumination is shown in Fig. 4. The corresponding distribution in the pure NW array with *H* and *D* of 2000 and 500 nm is also presented for comparison. It is obvious that the distribution region of photo-generated carriers is expanded owing to the synergetic effect of enhanced antireflection and light scattering after introducing the appropriate top modification. It is consistent with the boosted ^N^*J*_ph_/enhanced light confinement for the modified arrays, as exhibited in Fig. 2b. Moreover, the expansion of the photo-generated carrier distribution is beneficial for carrier collection especially for the planar *pn* junction configuration, and meanwhile makes the structures more tolerable to bulk defects/poor material qualities. It is worth noting that compared to the pure NW array, top modification also leads to the remarkably increased carrier density on the surface, and surface passivation is necessary to reduce surface recombination losses of photo-generated carriers for such arrays [66, 67].

**Fig. 5:**
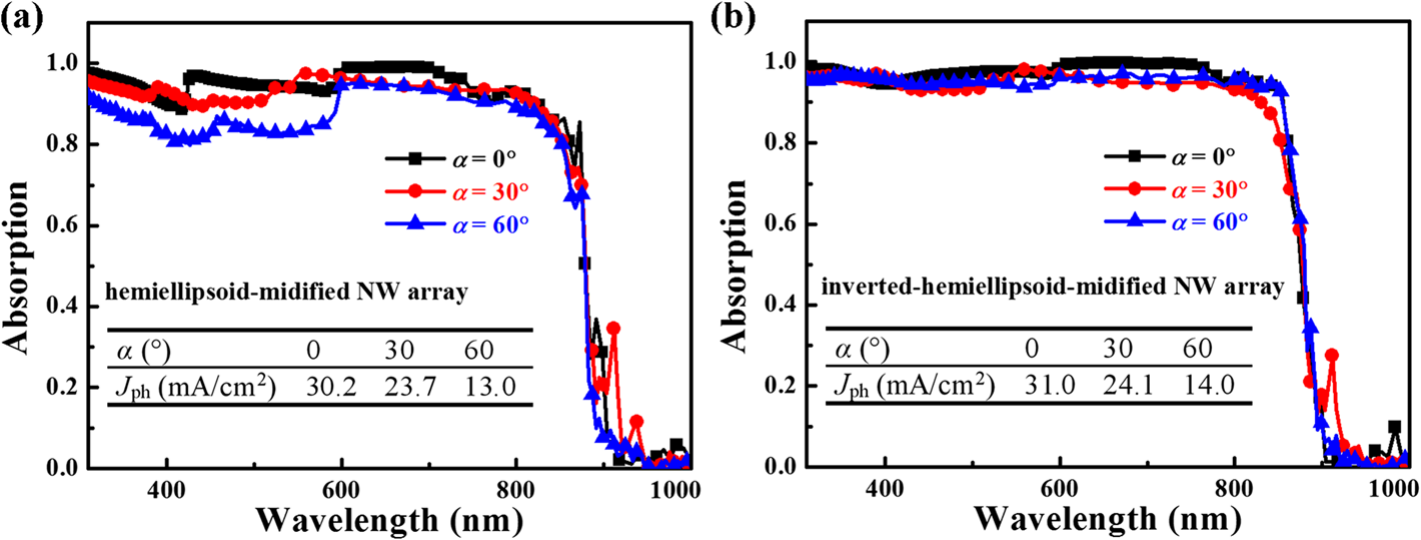
Absorption spectra of the **a** hemiellipsoid- and **b** inverted hemiellipsoid-modified GaAs NW arrays (*H* = 2000 nm, *D* = 500 nm, and *h* = 500 nm) at the incident angle (*α*) of 0, 30, and 60°. The inset tables summarize the theoretical photocurrent density (*J*_ph_) for these two top-modified NW arrays at the corresponding incident angles, respectively

As an excellent light absorber, effective light trapping under oblique incidence is necessary. Figure 5 exhibits the absorption spectra at the incident angle, *α* = 0, 30 and 60 degrees (°) for the (a) hemiellipsoid- and (b) inverted hemiellipsoid-modified GaAs NW arrays with the same structural parameters to the arrays shown in Fig. 4. It is remarkable that even at *α* = 60°, only limited degradation is observable, indicating excellent omnidirectional light confinement by both modifications. The calculated photocurrent density, *J*_ph_ for these two arrays is summarized in the inset of Fig. 5a and b. One notes that compared to *J*_ph_ of ~ 27.7 and 16.0 mA/cm^2^ for an ideal GaAs absorber at *α* = 30° and 60°, respectively, the corresponding value for both modified NW arrays only shows limited reduction.

**Fig. 6:**
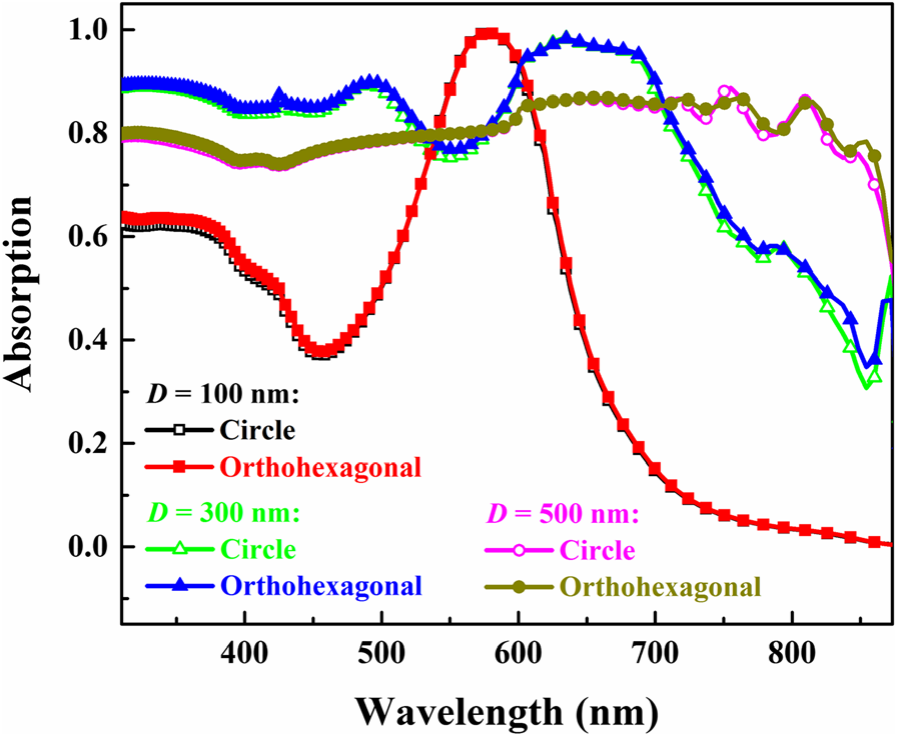
Comparison of the absorption spectra of the GaAs pure NW arrays with the circle and orthohexagonal wire cross-sections. The array period and wire length are 600 nm and 2 μm, respectively. The wire volumes for the corresponding NW arrays are the same and characterized by the diameter (100, 300, and 500 nm) of the NWs with a circle cross-section

It is known that for experimentally fabricated NWs, the surfaces are normally not such smooth like the ones adopted in the simulations. To check the validity of the simulation results for guiding experimental study, optical characteristics of the GaAs NW arrays with an orthohexagonal wire cross-section were simulated and compared with that of the corresponding NW arrays with a circle wire cross-section. Figure 6 compares the absorption spectra of these two kinds of arrays with the same volume (characterized by the diameter (100, 300 and 500 nm) of the circle NWs) and wire length of 2 μm in the spectral range of 310 nm (4 eV) to 873.2 nm (1.42 eV, i.e., the bandgap energy of GaAs). One notes that there are no evident differences of the optical behaviors between these two kinds of NW arrays in the considered spectral range. Accordingly, it is believed that the simulation results concluded from the NW arrays with a circle wire cross-section are also applicable to other arrays with a different wire cross-section.

Moreover, from the above discussion, it is evidenced that combination of the top modification for spatial modulation of the refractive index and enhanced light scattering by the bottom structure with matched characteristic dimension is an easily operated guideline for guiding design of high-performance light absorbers.

## Conclusions

In this paper, top modification of semiconductor nanowires using hemiellipsoids and inverted hemiellipsoids is introduced for further improving light confinement in the corresponding arrays. Systematic investigation unveils that high performance light management at limited effective thicknesses can be realized owing to the synergetic effect of improved antireflection and light scattering after introducing appropriate modification. For example, the inverted hemiellipsoid-modified GaAs nanowire array can trap 90% and 95% of the incident photons with the energy larger than the bandgap energy at the effective thickness of only ~ 180 and 270 nm. It is found that the top-modified NW arrays exhibit excellent light trapping capability in a broad range of the modification height. Meanwhile, spatial distribution of the photo-generated carriers is expanded for the modified nanowire arrays compared to the corresponding one without top modification, further indicating the improved light management. It would facilitate carrier collection, especially for the planar *pn* junction configuration. Moreover, further study indicates that the modified optical structures exhibit excellent omnidirectional light confinement, as expected for advanced light absorbers.

## Abbreviations

*J*_ph_: photocurrent density
^N^*J*_ph_: normalized theoretical photocurrent density
NW: nanowire
PV: photovoltaic
